# Quantifying the Transition from Unconscious to Conscious Detection of Temporal Patterns in Vigilance Tasks: A Unique Adaptation of Mackworth’s Clock Test

**DOI:** 10.3390/neurolint16050071

**Published:** 2024-08-31

**Authors:** Guaraci Lima de Morais, Tatiana Okubo Rocha Pinho, Leonardo Crespim, Osmar Pinto Neto

**Affiliations:** 1 Biomedical Engineering Department, Anhembi Morumbi University, São José dos Campos 12247-016, SP, Brazil; guaraci.morais@fatec.sp.gov.br; 2 Faculdade de Tecnologia de São Paulo, Campus Prof. Jesen Vidal, São José dos Campo 12247-016, SP, Brazil; 3 Arena235 Research Lab., São José dos Campos 12246-876, SP, Brazil; tatiokubo@gmail.com (T.O.R.P.); leocrespim@gmail.com (L.C.); 4 Gulfstream Aerospace, Savannah, GA 31408, USA; 5 Department of Kinesiology, California State University San Marcos (CSUSM), San Marcos, CA 92096, USA; 6 Center of Innovation, Technology and Education—CITÉ, São José dos Campos 12247-016, SP, Brazil

**Keywords:** implicit learning, vigilance, pattern recognition, Mackworth’s Clock Test, reaction time, conscious and unconscious detection

## Abstract

This study investigates the cognitive mechanisms underlying vigilance and pattern recognition using a novel adaptation of Mackworth’s Clock Test. We aimed to quantify the time it takes for temporal patterns detected unconsciously through implicit learning to surface in the conscious mind within a dynamic vigilance task environment. Forty-eight participants detected random and non-disclosed rhythmic anomalous clock hand movements in this setting. Our results indicate significant variability in detection accuracy, reaction times, and the ability to recognize the hidden pattern among participants. Notably, 23% of all participants and 56% of those who consciously reported the pattern exhibited statistically lower reaction times indicative of knowledge of the pattern 40 s before conscious identification. These findings provide valuable insights into the transition from unconscious to conscious detection, highlighting the complexity of sustained attention and pattern recognition. The study’s implications extend to designing training programs and tasks for high-stakes professions requiring prolonged vigilance. Future research should further explore the cognitive and neural correlates of these processes and the impact of task complexity on performance.

## 1. Introduction

The challenge of quantifying the time it takes for a temporal pattern detected unconsciously to surface in the conscious mind is significant yet underexplored. Understanding this transition is particularly important in contexts where sustained attention, or vigilance, plays a critical role, such as in air traffic control, surveillance, and quality inspection. Several studies have addressed related questions, emphasizing the distinction between conscious and unconscious processing. Dehaene and colleagues [1] discussed how unconscious processing could precede conscious awareness, suggesting a multi-stage cognitive mechanism. Similarly, studies on implicit learning have shown that individuals can learn complex patterns without being explicitly aware [2,3,4]. Moreover, research in artificial grammar learning (AGL) has demonstrated that people can extract and use knowledge structured by complex regularities, even when unaware [4,5,6]. This phenomenon, known as implicit or unconscious learning, plays a crucial role in human behavior across various contexts, including social interactions, sports, and those requiring sustained vigilance [7,8,9].

Most studies showing evidence of implicit learning have been conducted in experimental paradigms with relatively simple, artificial stimuli. For example, in AGL, participants are typically exposed to meaningless letter strings structured by artificial grammar [6,10]. In visuomotor sequence learning tasks, participants learn regularities in determining the location of a simple stimulus [11]. These stimuli and learning contexts differ significantly from those encountered in ecological situations, raising questions about the generalizability of these findings to real-life contexts. Characteristics of surface stimuli, such as their familiarity and ecological relevance, significantly influence the amount of implicit learning [12,13].

There have been attempts to use more realistic, socially relevant stimuli in implicit learning. Norman and Price [6] used sequences of yoga posture images and individually presented letters to show that sequences of body movements are learned less explicitly than letter sequences. Similarly, Ziori and Dienes [13] demonstrated implicit learning of face sequences. Zhang et al. [14] used point-light displays showing biological movements to investigate implicit learning of movement sequences, finding that participants could learn sequences showing inversion symmetry. Recent studies have expanded on these findings by exploring complex motor skills and gait patterns. For instance, Jack et al. [15] developed a task probing implicit learning of kinematically complex multi-articular movements, revealing that participants could implicitly learn repeating trajectories embedded within random ones. Additionally, Duppen et al. [16] investigated implicit and explicit motor learning during gait training with distorted rhythmic auditory cues, showing that participants adjusted their gait patterns to subtle changes in metronome frequency, even without conscious awareness. Furthermore, Jurchiș and Dienes [17] explored whether people could implicitly learn regularities from dynamic stimuli showing human movement using schematic patterns of point-light displays in an immersive virtual reality environment. Despite these advances, the path taken in their study differs from ours, as we focus on capturing the transition from unconscious to conscious detection over extended periods using a continuous task in the context of a dynamic vigilance environment.

Another challenge in studying implicit learning is determining whether the acquired knowledge is unconscious. Theories of consciousness generally agree on the reportable nature of conscious knowledge [18,19,20]. Most consciousness researchers advocate using subjective methods to assess whether participants possess conscious knowledge [21,22]. A common approach is to analyze participants’ accuracy in trials where they claim to have no conscious knowledge, with above-chance accuracy indicating unconscious knowledge [23,24,25]. However, misclassifying conscious trials as unconscious due to measurement errors remains a concern [26,27].

Our study presents an innovative adaptation of Mackworth’s Clock Test [28] to investigate the transition from unconscious to conscious detection of complex temporal patterns. The Mackworth Clock Test, developed by Norman Mackworth in 1948, is a classic experimental paradigm designed to study vigilance and sustained attention. In this test, participants are asked to observe the continuous movement of a clock’s hand, which occasionally makes an unexpected jump, deviating from its regular motion. The task requires participants to detect these irregular jumps, measuring their ability to maintain focused attention over prolonged periods. The test has been widely used to investigate the decline in vigilance over time and the cognitive processes involved in monitoring infrequent events.

Unlike previous studies focusing on isolated stimuli, our approach involves a continuous task where participants detect patterns in the timing of an object’s movement. This design allows us to capture the transition from unconscious to conscious detection over extended periods while assessing vigilance performance. We hypothesize that participants will unconsciously detect complex temporal patterns, becoming consciously detectable after repeated exposure. Specifically, we expect that the transition from unconscious to conscious detection will be marked by decreased reaction time as participants become more familiar with the pattern. Additionally, we hypothesize that individual differences in the ability to detect and report these patterns will exist and that vigilance may play a role in detection performance.

## 2. Materials and Methods

### 2.1. Participants

The study involved 48 voluntary participants from a Technology College in São José dos Campos, SP, with formal authorization and ethics approval (CAAE: 67190123.3.0000.5492). Participants were all over 18 years of age and provided prior agreement to the Informed Consent Form (ICF). The Universidade Anhembi Morumbi Ethics Committee approved the study protocol, CAAE: 67190123.3.0000.5492.

### 2.2. Task Description

The study employed a novel adaptation of Mackworth’s Clock Test to investigate participants’ ability to detect anomalous movements unconsciously [28]. The base code was created in PsyToolkit [29,30] and implemented using a custom-designed script that altered the clock’s behavior. The code was translated to HTML to run on a proprietary online server, recording personal information and the participants’ responses.

### 2.3. Experimental Design and Task Implementation

**Figure 1:**
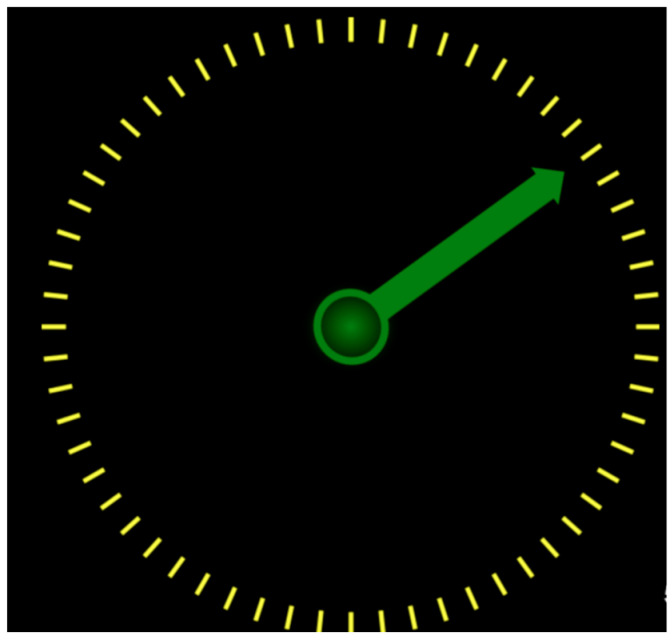
Diagram of the task setup: the visual representation of the Mackworth Clock Test setup, showing the clock face, clock circle, and clock hand.

The task was designed to run in full-screen mode with various visual elements, including instructions, strategy prompts, and the clock interface (clock face, clock circle, and clock hand, Figure 1).

Participants observed the clock hand movement for a total of 500 s, or approximately 8 min. Clock movement was divided into two distinct phases that varied in how the clock hand moved: an initial random phase with a duration of 150 s of duration and a subsequent patterned phase with a duration of 350 s.

During the first 150 s, or random phase, the clock hand mostly moved in discrete steps of 6 degrees; however, sometimes, at random, the clock hand moved 24 degrees. We introduced this anomaly by assigning a 10% probability per tick that the clock hand would step anomalously, ensuring that anomalies were sporadic and unpredictable. The script was designed to prevent the same anomaly from repeating consecutively. It is important to note that because anomalies were determined using a 10% probability of happening for every clock tick, the number of anomalous movements varied between participants in the first 150 s.

After the initial random phase, the task transitioned into a patterned phase that lasted until the task ended at 500 s. During this phase, as in the random phase, the clock hand mostly ticked in discrete steps of 6 degrees. However, in this phase, anomalies followed a fixed pattern to allow for the investigation of pattern recognition. The pattern consisted of nine regular clock hand movements (6 degrees) followed by one anomalous movement (24 degrees), four regular movements followed by one anomalous movement, nine followed by one, then four followed by one, and so on. Unlike the random phase, the number of anomalous movements was the same across participants during the second portion of the task, and the anomaly followed a fixed pattern.

### 2.4. Task Flow and Data Collection

Participants were required to complete both phases of the task, with the flow as follows:

- Clock Display: The clock face and hand were displayed, with the hand updating its position based on the defined rules.
- Anomaly Detection: Participants pressed a designated key if they detected an anomaly. Responses, including reaction time (RT) and the correctness of the detection, were recorded.
- Feedback Mechanism: Positive feedback (green circle) was given for correct anomaly detections, while negative feedback (red circle) was provided for false or missed anomalies.
- Pattern Report: Participants were instructed to click a button when they believed they had identified a pattern in the anomalous movements of the clock hand. Upon clicking, the task paused, and a prompt asked them to describe the pattern they detected. If their description matched the actual pattern, the time of their click was recorded as “Reported Instant”. This timestamp allowed us to analyze the transition from unconscious detection to conscious awareness.
- Data Recording: Data, including hand position, reaction time, and detection correctness, were recorded each second and saved.

### 2.5. Data Analysis

The analysis was performed using MATLAB 2022a. We recorded the total count of correctly detected anomalies (Number of Correct Detections), incorrect responses where no anomaly was present (Number of Incorrect Detections), and anomalies that were not detected (Number of Missed Detections). We also determined the RT mean and standard deviation at the random and patterned portion of the task, and the instant participants correctly reported detecting a pattern.

Furthermore, we used the reaction times (RTs) from the random phase of the clock task to establish a threshold for determining whether an RT was significantly lower than expected, potentially indicating a reaction time effort consistent with either implicit or explicit knowledge of when the anomaly was going to happen. To achieve this, we fit a normal distribution to the RT data points from the random phase and employed Monte Carlo simulations with 10,000 iterations to estimate the lower 5% threshold. This threshold is commonly used in statistical analyses to reject the null hypothesis and serves as a conservative estimate for detecting deviations that may suggest unconscious processing.

For each subject, we identified all RTs below this threshold (referred to as Unconscious RT) during the patterned phase of the task, excluding the first 17 s (which represents the first cycle and a half of the pattern) and before the time when the subject reported consciously detecting the pattern, for those who did so. We calculated the mean and standard deviation of these Unconscious RTs and defined the moment of unconscious detection as the earliest occurrence of an RT below the threshold. For those who successfully identified the pattern, the duration from this moment of unconscious detection to the subject’s conscious report of the pattern was then used to determine the transition time from unconscious to conscious detection.

### 2.6. Statistics

Statistical analysis was carried out in Visual Studio Code (v. 1.92.1) using Python (v. 3.12). We used the Smirnov–Kolmogorov Normality test to verify the normality of data and choose between parametric and non-parametric tests. We compared correct, incorrect, and missed detections from the participants using one-way ANOVA and paired *t*-tests with Bonferroni corrections. We also compared participants who found and did not find patterns across the different measurements with *t*-tests and Mann–Whitney tests. Depending on the normality results, we investigated the correlations between variables using Pearson’s or Spearman’s methods. To confirm our choice of unconscious RT threshold, we also compared, using independent *t*-tests, the random and unconscious RT values from each subject.

## 3. Results

Four participants were excluded from the analysis because they had only 0, 1, or 2 correct detections, which suggests they either did not understand the task or had other issues related to completing it.

### 3.1. Conscious and Unconscious Detection

The analysis aimed to estimate the time for unconscious detection to transition to conscious awareness. Our results indicated that out of 44 participants who completed the task successfully, 20 (45%) demonstrated statistically low RT during the task’s pattern phase before reporting the pattern, suggesting a unconscious “knowledge” of the instant of occurrence of the anomalous clock movements, i.e., implicit learning. The mean threshold determined was 448.81 ± 164.2 ms. The mean instant of first unconscious detection for all 20 participants who exhibited implicit learning was 222.60 ± 45.71 s. Participants exhibited multiple reaction times below the threshold—on average, 5.7 ± 2.2 times.

To double check the statistical reliability of the chosen threshold, we compared each subject’s random RT to their unconscious RT. We found that the RTs were significantly different for 20 of 22 (91%) participants (*p*-value of 0.02 ± 0.03 for all participants).

Out of the 20 participants who demonstrated implicit learning, only 10 (50%) eventually accurately reported that they had found the pattern, and the duration from unconscious to conscious was 41.0 ± 29.36 s.

Table 1 summarizes the results and shows the mean and standard deviation (Std) of the study’s parameters for all participants, divided among participants who could and could not accurately report the task pattern.

### 3.2. Pattern Recognition

**Figure 2:**
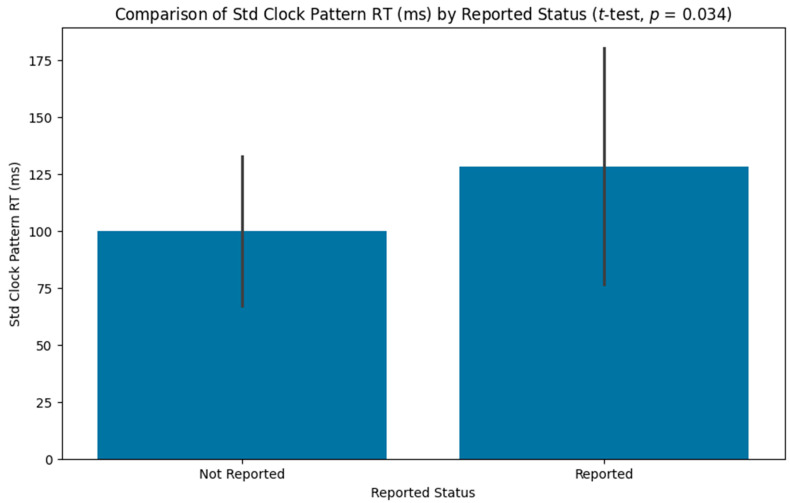
Bar graph comparing the reaction time variability during the pattern phase between participants who accurately identified the pattern and those who did not.

Of the 44 participants who completed the task, 18 (41%) accurately identified the pattern of anomalous movements in the clock task. The results indicate that the reaction time variability during the pattern portion of the task was significantly (*p* = 0.034) higher for the people who accurately reported the pattern (128.48 ± 52.01) compared to those who did not (99.94 ± 32.77; Figure 2).

### 3.3. Detection Performance and Vigilance

Although not our primary focus, our adaptation of the Mackworth Clock Test allowed us to assess participants’ vigilance and yielded insightful results. Out of the 44 participants who completed the task, only 3 (7%) had a perfect score, i.e., 0 incorrect and missed detections. Participants correctly detected 78.36 ± 20.3% of valid clock anomalous movements on average. Correct, incorrect, and missed detections were 36.86 ± 9.92, 6.0 ± 5.65, and 10.2 ± 9.73, respectively; correct detections were significantly (*p* < 0.001) greater than both incorrect and missed detections, and missed detections were significantly (*p* < 0.0.001) greater than incorrect detections.

Furthermore, we found that correct detections were significantly negatively correlated to incorrect (Spearman ρ = −0.68, *p* < 0.001) and missed detections (Spearman ρ = −0.82, *p* < 0.001). Finally, incorrect detections were significantly correlated to missed detections (Spearman ρ = 0.72, *p* < 0.001).

### 3.4. Reaction Time Analysis

The mean RT for the random portion of the clock task was 640.99 ± 122.62 ms, while the mean variability of the random RT was 116.8 ± 58.21 ms. Additionally, the mean RT for the pattern portion of the task was 597.22 ± 141.9, and its variability was 115.04 ± 47.02 ms.

**Figure 3:**
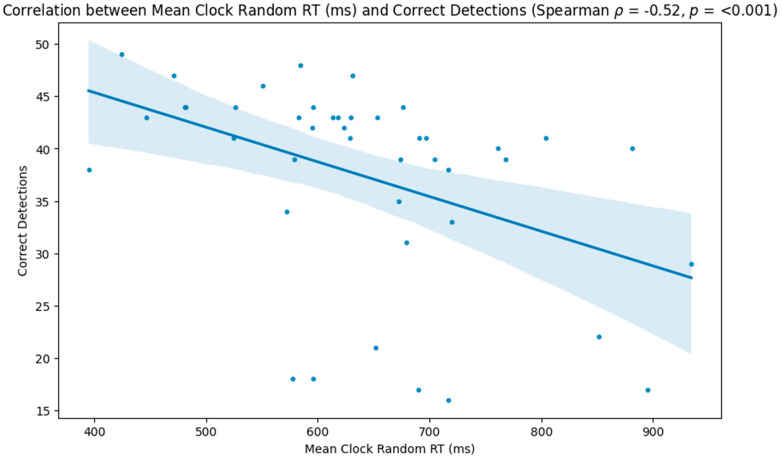
Scatter plot showing the relationship between participants’ reaction times during the random phase of the task and the number of correct detections.

**Figure 4:**
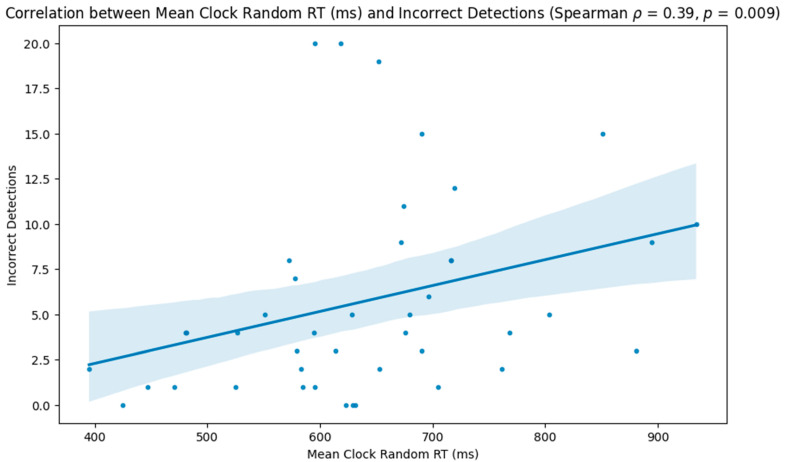
Scatter plot showing the relationship between participants’ reaction times during the random phase of the task and the number of incorrect detections.

**Figure 5:**
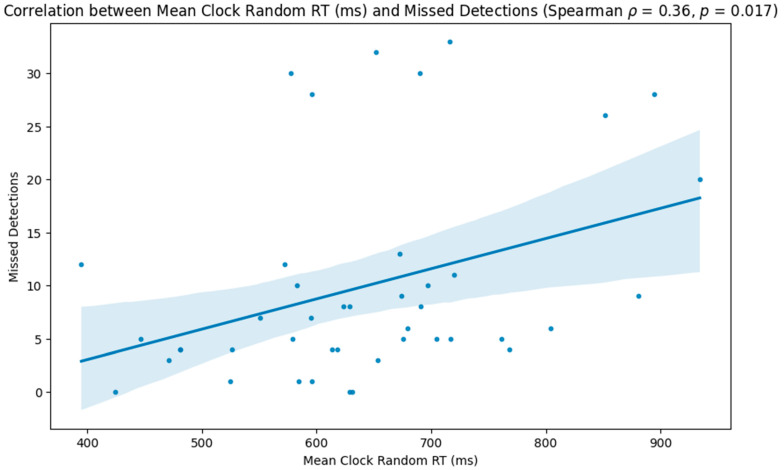
Scatter plot showing the relationship between participants’ reaction times during the random phase of the task and the number of missed detections.

The results indicated that participants with lower reaction times on the random portion of the task performed better, with higher correct detections (Spearman ρ = −0.52, *p* < 0.001; Figure 3) and lower incorrect (Spearman ρ = 0.39, *p* = 0.009; Figure 4) and missed detections (Spearman ρ = 0.36, *p* = 0.017; Figure 5).

## 4. Discussion

The results of this study provide significant insights into the mechanisms underlying vigilance and detection of random and patterned anomalies. By employing a novel adaptation of Mackworth’s Clock Test [28], we could examine participants’ ability to detect irregularities and recognize structured patterns over an extended period. This design allowed us to focus on the time it takes for unconsciously detected patterns to become consciously recognized. Overall, the results demonstrate that 41% of the participants (18/44) could accurately detect the pattern of anomalies with varying degrees of accuracy and response consistency. Additionally, 45% of all participants (20/44) exhibited implicit learning. Of these 20 participants only 10 correctly identified the pattern, in other words, the other 10 participants exhibited implicit learning but did not consciously find the pattern. For the 10 participants who demonstrated implicit learning and were able to accurately report the pattern, the duration between the first instance they exhibited implicit learning to the instance they reported the pattern was on average 41 s. This study provides valuable insights into the conscious and unconscious detection processes involved in vigilance tasks.

### 4.1. Pattern Recognition

One of the primary findings of this study is that 41% of the participants accurately identified the pattern of anomalies. This indicates that while a substantial proportion of participants could detect and report structured patterns, many struggled with this task. The variability in performance highlights individual differences in vigilance and pattern recognition abilities, consistent with previous research on sustained attention [31]. Our findings suggest that vigilance, assessed through detection accuracy and reaction times, plays a critical role in transitioning from unconscious to conscious pattern recognition. The increased reaction time variability among participants who accurately identified the pattern may reflect the cognitive load of maintaining vigilance while processing complex temporal information. Additionally, it is possible that once a subject actively perceived the pattern, they did not strive to click the mouse as quickly as possible, knowing when the anomaly would occur. In contrast, we found no significant differences in other measured parameters when comparing participants who accurately detected the pattern to those who did not (*p* > 0.10). This lack of substantial differences suggests that the ability to detect and report the pattern may not solely depend on attention-related performance metrics but could involve other cognitive factors such as memory and pattern recognition skills.

### 4.2. Choice of Threshold

Our results indicated that the choice of a 5% threshold for identifying unconscious detection was statistically sound, as demonstrated by the significant differences observed between random and unconscious reaction times. However, we acknowledge that determining an appropriate threshold remains one of the more challenging aspects of our methodology. While our approach was individualized, using each participant’s reaction times during the random phase to set the threshold, the use of a fixed 5% cut-off may still have limitations in fully capturing the nuances of unconscious processing across all participants. Future studies should consider whether other methods, such as dynamic or context-dependent thresholds, might provide a more precise measure of unconscious detection. Additionally, further research could examine the implications of different threshold levels on the detection of unconscious cognitive processes to refine and enhance the robustness of this approach.

### 4.3. Implicit Learning: Conscious and Unconscious Detection

A crucial aspect of our study was the investigation of unconscious detection and its transition to conscious awareness. We found that 45% of the participants exhibited implicit learning, and that 50% of them were able to accurately report the pattern. Furthermore, our results suggest that, for those who could accurately report the pattern, it took on average 41 ± 29 s from the moment they first exhibited implicit learning to the moment they reported the pattern. This finding aligns with previous research suggesting that unconscious processing can precede conscious awareness [1]. Moreover, recent studies on implicit learning have demonstrated that the brain can process and respond to patterns without conscious awareness, further supporting our observations [17,32].

Our findings indicate that unconscious knowledge can influence behavior before transitioning into conscious awareness. This is consistent with the work of Norman et al. (2019) [33], who found that participants could utilize structural knowledge of an artificial grammar without being consciously aware of it. Similarly, Dienes and Scott (2005) [5] demonstrated that individuals can apply complex regularities unconsciously, suggesting that unconscious structural knowledge can be strategic and impactful.

However, the fact that no clear evidence of implicit learning was found for 55% of the participants indicates that this phenomenon may be more nuanced and individualized. Factors such as familiarity with the stimuli [10] and the ecological relevance of the task [12,13] might influence the effectiveness and detectability of unconscious learning. Previous research has highlighted the complexity and variability of implicit learning mechanisms, suggesting that individual differences play a significant role in how unconscious detection transitions to conscious awareness [34,35,36].

Further research is needed to elucidate the conditions under which unconscious detection reliably transitions to conscious awareness and to explore the cognitive and neural mechanisms underlying these processes. Advances in neuroimaging techniques, such as fMRI and EEG, could provide deeper insights into the brain regions and networks involved in these transitions [21,23]. Additionally, future studies should consider the role of task complexity and environmental factors in modulating unconscious detection, as these elements are critical for understanding how implicit learning operates in real-world settings [8,9].

### 4.4. Detection Performance and Vigilance

Although not the primary focus, our adaptation of Mackworth’s Clock Test allowed us to assess participants’ vigilance, yielding insightful results. The correlation between reaction times during the random phase and detection performance indicates that vigilance significantly impacts participants’ ability to detect anomalies. Participants with quicker reaction times were more accurate in detecting anomalies, suggesting that sustained attention is crucial for optimal performance in this task. This finding underscores the importance of vigilance in the context of our study. It aligns with our goal of understanding the cognitive processes that support the transition from unconscious to conscious detection. Our results demonstrate how complex attention tasks such as the Mackworth Clock Test are, as only 7% of the participants had a perfect score. Nevertheless, on average, participants correctly detected 78.36 ± 20.3% of the anomalies, which shows its efficient test range. Furthermore, we found that variability in attention can be seen in all three measurements, as they all correlate.

The reaction time analysis from the random portion of the task revealed significant variability among participants (mean RT = 640.99 ± 122.62 ms). This variability suggests differences in processing speed and response consistency. Strikingly, this variability in RT correlated with the variability in participants’ ability to perform the task accurately. Participants with lower reaction times exhibited higher correct detections and lower incorrect and missed detections, suggesting that faster processing speeds may enhance vigilance performance.

### 4.5. Implications and Future Directions

The findings of this study have several practical implications. Understanding the mechanisms of vigilance and pattern recognition can inform the design of tasks and training programs to improve performance in high-stakes professions requiring sustained attention. For example, tailored training interventions that enhance individuals’ ability to detect patterns and maintain vigilance over long periods could benefit air traffic control, surveillance, and quality inspection. These fields often rely on rapid and accurate detection of subtle anomalies, making our insights particularly valuable.

Future research should explore the cognitive and neural mechanisms underlying individual vigilance and pattern recognition differences. Neuroimaging techniques such as functional MRI (fMRI) and electroencephalography (EEG) could provide deeper insights into the brain regions and networks involved in these processes [21]. Identifying the neural correlates of unconscious and conscious detection could reveal the transition mechanisms between these states, contributing to a more comprehensive understanding of cognitive processing.

Additionally, longitudinal studies examining how training and practice influence vigilance and pattern recognition over time would be valuable. These studies could help determine the long-term effects of different training regimens on the ability to detect patterns and maintain vigilance. Another potential direction for future research is to investigate the role of task complexity and environmental factors on vigilance performance. By varying the difficulty and context of vigilance tasks, researchers can better understand how these factors influence detection accuracy and response times. This knowledge can be applied to optimize task design and create more effective training programs, enhancing performance in real-world settings.

### 4.6. Limitations

While this study provides valuable insights, it is essential to acknowledge its limitations. The sample size, although adequate, may not fully capture the variability in vigilance performance across different populations. Additionally, using a single type of vigilance task limits the generalizability of the findings to other types of vigilance tasks. Future studies should include larger, more diverse samples and a variety of task designs to enhance the robustness of the findings. Moreover, the specific parameters of our task may not fully encompass all aspects of real-world vigilance tasks. Further research should aim to replicate our findings across different task paradigms and settings to validate the generalizability of our results.

## 5. Conclusions

In conclusion, this study advances our understanding of vigilance and pattern recognition by employing an innovative adaptation of Mackworth’s Clock Test. Our findings highlight the variability in individuals’ ability to detect and report structured patterns and underscore the complexity of unconscious and conscious detection processes. The results demonstrate that many participants can accurately detect patterns, with many showing signs of unconscious detection before conscious awareness of approximately 40 s. These insights are essential for designing tasks and training programs to improve vigilance performance in various professional settings.

Future research should continue to explore the cognitive and neural mechanisms underlying vigilance and pattern recognition and the factors influencing these critical abilities. Additionally, further studies should investigate alternative methods for determining unconscious detection thresholds and consider the role of task complexity and environmental factors in vigilance performance. By addressing these areas, researchers can develop more effective strategies for enhancing vigilance and pattern recognition, ultimately improving performance in high-stakes environments.

## Figures and Tables

**Table 1:** Mean and standard deviation (Std) of the study’s parameters for all participants (All) and divided among participants who could (Reported) and could not (Not Reported) accurately report the task pattern.

Variables	Mean (All)	Std (All)	Mean (Reported)	Std (Reported)	Mean (Not Reported)	Std (Not Reported)
Correct Detections	36.86	9.92	37.35	9.88	36.33	10.17
Incorrect Detections	6.00	5.65	5.04	4.46	7.05	6.68
Missed Detections	10.20	9.73	10.00	9.66	10.43	10.05
Mean Clock Random RT (ms)	640.99	122.62	637.84	124.00	644.61	135.20
Std Clock Random RT (ms)	116.80	58.21	104.80	49.60	135.20	67.02
Mean Clock Pattern RT (ms)	597.22	141.90	586.08	164.50	612.29	96.86
Std Clock Pattern RT (ms)	115.04	47.02	128.48	52.01	96.86	32.53
Reported Instant (s)			273.28	54.68		
Threshold (ms)	448.81	164.20	451.84	142.98	445.49	188.29
Instant First Unconscious Detection (s)	222.60	45.71	227.80	41.06	217.40	51.62
Duration Unconscious to Conscious (s)			41.00	29.36		
Mean Unconscious RT (ms)	447.35	193.00	409.23	144.76	518.16	258.92
Std Unconscious RT (ms)	34.48	52.83	42.99	63.30	18.68	19.40
p-value Random vs. Unconscious RT	0.020	0.031	0.010	0.013	0.039	0.044

## Data Availability

The data supporting this study’s findings are available from the corresponding author, O.P.N., upon reasonable request.

## References

[1] Dehaene S., Changeux J.P., Naccache L., Sackur J., Sergent C. (2006). Conscious, preconscious, and subliminal processing: A testable taxonomy. Trends Cogn. Sci..

[2] Cleeremans A., Destrebecqz A., Boyer M. (1998). Implicit learning: News from the front. Trends Cogn. Sci..

[3] Dienes Z., Seth A.K. Chapter 6 Consciousness: Conscious versus Unconscious Processes. https://sussex.figshare.com/articles/chapter/Consciousness_conscious_versus_unconscious_processes/23419916.

[4] Reber A.S. (1967). Implicit learning of artificial grammars. J. Verbal Learn. Verbal Behav..

[5] Dienes Z., Scott R. (2005). Measuring unconscious knowledge: Distinguishing structural knowledge and judgment knowledge. Psychol. Res..

[6] Norman E., Price M.C. (2012). Social intuition as a form of implicit learning: Sequences of body movements are learned less explicitly than letter sequences. Adv. Cogn. Psychol..

[7] Costea A.R., Jurchiș R., Visu-Petra L., Cleeremans A., Norman E., Opre A. (2023). Implicit and explicit learning of socio-emotional information in a dynamic interaction with a virtual avatar. Psychol. Res..

[8] Raab M., Johnson J.G., Plessner H., Betsch C., Betsch T. (2008). Implicit learning as a means to intuitive decision making in sports. Intuition in Judgment and Decision Making.

[9] Weiss S.M., Reber A.S., Owen D.R. (2008). The locus of focus: The effect of switching from a preferred to a non-preferred focus of attention. J. Sports Sci..

[10] Scott R.B., Dienes Z. (2010). Prior familiarity with components enhances unconscious learning of relations. Conscious Cogn..

[11] Nissen M.J., Bullemer P. (1987). Attentional requirements of learning: Evidence from performance measures. Cognit. Psychol..

[12] Eitam B., Glass-Hackel R., Aviezer H., Dienes Z., Shoval R., Higgins E.T. (2014). Are task irrelevant faces unintentionally processed? Implicit learning as a test case. J. Exp. Psychol. Hum. Percept. Perform..

[13] Ziori E., Dienes Z. (2015). Facial beauty affects implicit and explicit learning of men and women differently. Front. Psychol..

[14] Zhang Q., Li L., Guo X., Zheng L., Wu Y., Zhou C. (2020). Implicit learning of symmetry of human movement and gray matter density: Evidence against pure domain general and pure domain specific theories of implicit learning. Int. J. Psychophysiol. Off. J. Int. Organ. Psychophysiol..

[15] Jack P.S., Austin J.H., Sarah N.K., Tony G.J.I., Shaun G.B. (2024). A kinematically complex multi-articular motor skill for investigating implicit motor learning. Psychol. Res..

[16] Duppen C.P., Wrona H., Dayan E., Lewek M.D. (2024). Evidence of Implicit and Explicit Motor Learning during Gait Training with Distorted Rhythmic Auditory Cues. J. Mot. Behav..

[17] Jurchiș R., Dienes Z. (2023). Implicit learning of regularities followed by realistic body movements in virtual reality. Psychon. Bull. Rev..

[18] Liljenström H., Århem P. (2011). Consciousness Transitions: Phylogenetic, Ontogenetic and Physiological Aspects.

[19] Dienes Z., Rebuschat P., Williams J.N. (2011). Conscious versus unconscious learning of structure. Statistical Learning and Language Acquisition [Internet].

[20] Rosenthal D.M., Gennaro R.J. (2004). 2. Varieties of higher-order theory. Higher-Order Theories of Consciousness: An Anthology [Internet].

[21] Francken J.C., Beerendonk L., Molenaar D., Fahrenfort J.J., Kiverstein J.D., Seth A.K., Van Gaal S. (2022). An academic survey on theoretical foundations, common assumptions and the current state of consciousness science. Neurosci. Conscious.

[22] Rosenthal D. (2019). Consciousness and confidence. Neuropsychologia.

[23] Jurchiș R., Costea A., Dienes Z., Miclea M., Opre A. (2020). Evaluative conditioning of artificial grammars: Evidence that subjectively-unconscious structures bias affective evaluations of novel stimuli. J. Exp. Psychol. Gen..

[24] Newell B.R., Shanks D.R. (2014). Unconscious influences on decision making: A critical review. Behav. Brain Sci..

[25] Skora L.I., Livermore J.J.A., Dienes Z., Seth A.K., Scott R.B. (2023). Feasibility of unconscious instrumental conditioning: A registered replication. Cortex.

[26] Shanks D.R. (2017). Regressive research: The pitfalls of post hoc data selection in the study of unconscious mental processes. Psychon. Bull. Rev..

[27] Shanks D.R., Malejka S., Vadillo M.A. (2021). The Challenge of Inferring Unconscious Mental Processes. Exp. Psychol..

[28] Mackworth N.H. (1948). The Breakdown of Vigilance during Prolonged Visual Search. Q. J. Exp. Psychol..

[29] Stoet G. (2010). PsyToolkit: A software package for programming psychological experiments using Linux. Behav. Res. Methods.

[30] PsyToolkit: A Novel Web-Based Method for Running Online Questionnaires and Reaction-Time Experiments-Gijsbert Stoet. 2017. [Internet]. https://journals.sagepub.com/doi/abs/10.1177/0098628316677643.

[31] Parasuraman R., Warm J.S., See J.E. (1998). Brain systems of vigilance. The Attentive Brain.

[32] Guillemin C., Tillmann B. (2021). Implicit learning of two artificial grammars. Cogn Process.

[33] Norman E., Scott R.B., Price M.C., Jones E., Dienes Z. (2019). Can unconscious structural knowledge be strategically controlled?. Implicit Learning: 50 Years on.

[34] Perruchet P., Pacteau C. (1990). Synthetic grammar learning: Implicit rule abstraction or explicit fragmentary knowledge?. J. Exp. Psychol. Gen..

[35] Shanks D.R., St John M.F. (1994). Characteristics of dissociable human learning systems. Behav. Brain Sci..

[36] Maresch J., Mudrik L., Donchin O. (2021). Measures of explicit and implicit in motor learning: What we know and what we don’t. Neurosci. Biobehav. Rev..

